# DNA methylation of MMPs and TIMPs in atherothrombosis process in carotid plaques and blood tissues

**DOI:** 10.18632/oncotarget.27469

**Published:** 2020-03-10

**Authors:** Cristina Gallego-Fabrega, Natalia Cullell, Carolina Soriano-Tárraga, Caty Carrera, Nuria P. Torres-Aguila, Elena Muiño, Jara Cárcel-Márquez, Manuel Castro de Moura, Alba Fernández-Sanlés, Manel Esteller, Roberto Elosua, Jordi Jiménez-Conde, Jaume Roquer, Joan Montaner, Jerzy Krupinski, Israel Fernandez-Cadenas

**Affiliations:** ^1^Neurology, Hospital Universitari Mútua de Terrassa/Fundacio Docència i Recerca MutuaTerrassa, Terrassa, Spain; ^2^Stroke Pharmacogenomics and Genetics, Sant Pau Research Institute, Barcelona, Spain; ^3^Facultat de Medicina, Universitat de Barcelona, Barcelona, Spain; ^4^Department of Neurology, Hospital del Mar, Neurovascular Research Group, Institut Hospital del Mar d’Investigacions Mèdiques, Universitat Autònoma de Barcelona/DCEXS-Universitat Pompeu Fabra, Barcelona, Spain; ^5^Neurovascular Research Laboratory, Vall d'Hebron Institute of Research, Universitat Autònoma de Barcelona, Barcelona, Spain; ^6^Josep Carreras Leukaemia Research Institute (IJC), Badalona, Barcelona, Spain; ^7^Cardiovascular Epidemiology and Genetics Research Group, Hospital del Mar Medical Research Institute, Universitat Pompeu Fabra, Barcelona, Spain; ^8^Department of Physiological Sciences II, School of Medicine, University of Barcelona, Barcelona, Spain; ^9^Institucio Catalana de Recerca i Estudis Avançats, Barcelona, Spain; ^10^Centre for Biomedicine, Manchester Metropolitan University, Manchester, UK; ^*^These authors contributed equally to this work

**Keywords:** atherosclerotic plaque, epigenetics, DNA methylation, matrix metalloproteinases

## Abstract

Background and Purpose: Polymorphisms and serum levels of Matrix Metalloproteinases (MMP) and Tissue Inhibitor of Metalloproteinases (TIMP) have been studied with regard to atheromatous plaques and ischemic stroke, while no studies of DNA methylation (DNAm) patterns of *MMP* or *TIMP* have been performed to that end. Here, we evaluate DNAm levels of the MMP and TIMP gene families in human carotid plaques and blood samples of atherothrombotic stroke patients.

Methods: We profiled the DNAm status of stable and ulcerated atherosclerotic plaques obtained as pair sets from three patients who underwent carotid endarterectomy surgery. We selected 415 CpG sites, mapping into *MMPs* and *TIMPs* genes for further study. Secondly, the statistically associated CpG sites were analyzed in blood samples from two separate atherothrombotic stroke cohorts (total sample size = 307), ischemic stroke-cohort 1 (ISC-1): 37 atherothrombotic patients and 6 controls, ischemic stroke-cohort 2 (ISC-2): 80 atherothrombotic patients and 184 controls. DNAm levels from plaque tissue and blood samples were evaluated using a high-density microarray Infinium, HumanMethylation450 BeadChip and Infinium MethylationEPIC BeadChip.

Results: Three CpG sites were statistically significantly associated with unstable plaque portions; cg02969624, *q*-value = 0.035 (*TIMP2*), and cg04316754, *q*-value = 0.037 (*MMP24*) were hypermethylated, while cg24211657 *q*-value = 0.035 (*TIMP2*) was hypomethylated. Association of cg04316754 (*MMP24)* methylation levels with atherothrombotic risk was also observed in blood tissue: ISC-1 *p*-values = 0.03, ISC-2 *p*-value = 1.9 × 10^-04^.

Conclusions: The results suggest different DNAm status of MMP24 between stable and unstable atherothrombotic carotid plaques, and between atherothrombotic stroke and controls in blood samples.

## INTRODUCTION

Arteriosclerosis is the underlying pathology in most cases of cardiovascular disease (CVD), including ischemic stroke (IS), contributing to major mortality in Western countries. Atherosclerosis is a process that involves a complex interaction between different factors and cell types, including cells of the vessel wall and immune system. During their formation, atherosclerotic lesions undergo different stages, starting from inflammatory endothelial activation/dysfunction and resulting in plaque vulnerability and rupture [1].

Matrix Metalloproteinases (MMPs) are a family of zinc-binding proteolytic enzymes that are known for their ability to cleave one or more extracellular matrix constituents, as well as other proteins [2]. MMPs and their specific inhibitors, tissue inhibitors of metalloproteinases (TIMPs), are involved in many processes, including wound healing, angiogenesis, inflammation, and blood-brain barrier disruption [2, 3]. Uncontrolled expression of MMPs can result in tissue destruction and inflammation. MMPs and TIMPs have raised considerable interest within the atherosclerosis and IS research community, as they represent an attractive target for the use of current drugs and the development of novel ones, aimed at blocking MMP activity [4].

Some studies have observed associations between protein plasma levels of MMPs, TIMPs, atheromatous plaque instability [5, 6], stroke progression [7]. However, few studies have investigated the relationship between DNA methylation (DNAm) and atherosclerosis pathogenicity [8–10] and none have focused on the MMP and TIMP gene families. Zaina et al., observed widespread hypermethylation in the atherosclerotic portion of 15 aorta samples compared to healthy counterparts [9]. In a different study, Zaina et al., detected small DNAm changes between 19 symptomatic and asymptomatic plaque pairs, and a drift toward hypomethylation, associated with increasing post-cerebrovascular event time [8], whereas others identified a set of CpGs that drift toward hypermethylation with lesion progression [10], in 15 sample-pairs.

Here, we present a characterization of DNAm status of the *MMP* and *TIMP* gene families in donor-matched stable and ulcerated carotid artery atherosclerotic plaques and whole blood in atherothrombotic stroke patients and controls.

## RESULTS

**Figure 1:**
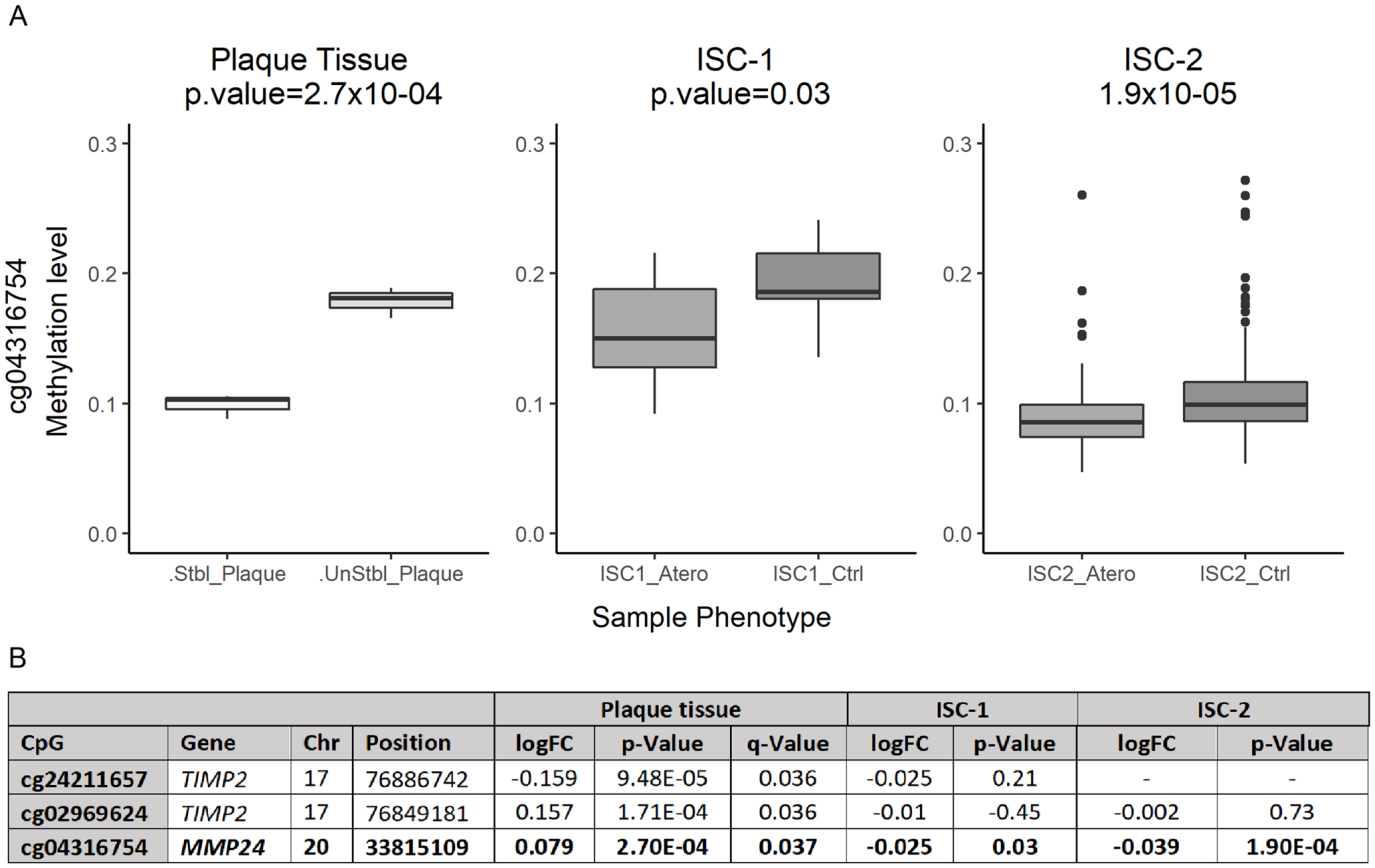
Differentially methylated levels of cg04316754 (*MMP24*). (**A**) Boxplot: differentially methylated status of cg04316754. X-axis indicates sample type, in each cohort. Y-axis indicates DNA methylation levels (β-values = 0–1).0 = 0% methylation status, 1 = 100% methylation status. (**B**) Statistically significant differentially methylated CpG sites in plaque tissue, annotation and statistical results in the three cohorts.

Seventy-two differentially methylated CpGs were observed between normal and ulcerated plaque portions (*p*-value < 0.05) (Supplementary Table 3). Sixty of them were hypermethylated in the ulcerated portion, and twelve were hypomethylated. Three CpGs passed FDR adjusted *p*-value (or *q*-value), two were hypermethylated: cg02969624, *q*-value = 0.036 mapping in *TIMP2*, and cg04316754, *q*-value = 0.037 mapping in *MMP24*; whereas cg24211657 *q*-value = 0.036 mapping in *TIMP2* was hypomethylated (Figure 1B) (Supplementary Table 4).

**Figure 2:**
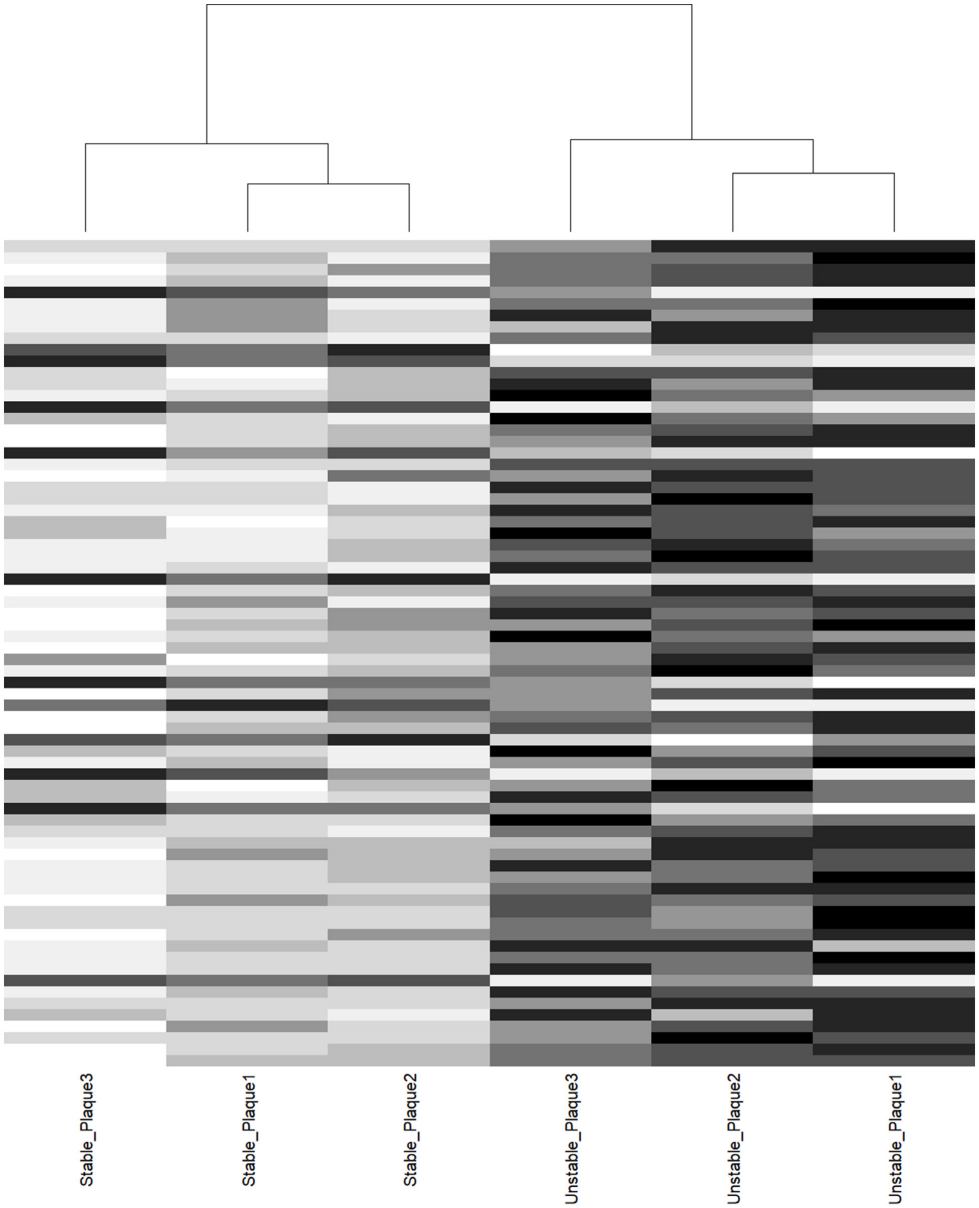
Cluster analysis of donor-matched aortic plaque samples. Supervised clustering of 72 CpGs nominal association. Columns represent samples, horizontal lines represent CpGs. Notice the perfect segregation between stable and unstable plaque counterpart.

Supervised clustering of the 72 significant sites distinguished all atherosclerotic lesions from their donor-matched healthy counterparts (Figure 2). Good clustering was also observed when using all 415 CpGs, highlighting the specificity of the MMPs and TIMPs in plaque progression discrimination (Supplementary Figure 2).

All CpGs were also interrogated in two independent blood sample ischemic stroke cohorts of atherothrombotic stroke subtype (TOAST classification), 415 CpGs in ISC-1 and 355 CpGs in ISC-2 (Supplementary Table 3). One of the significantly associated CpGs identified in the initial plaque study was also associated with the risk of atherothrombotic stroke. Thus, cg04316754, *MMP24*, was up-methylated in control samples when compared to cases in both cohorts. In ISC-1, we found a significant association of cg04316754 (Moldel_2 *p*-value = 0.025) that was also replicated in ISC-2 (Model_1 *p*-value = 1.9 × 10^-04^) (Supplementary Table 5) (Figure 1A). Thus, cg02969624 and cg24211657 (*TIMP2)* were up-methylated in control samples when compared to cases, but their association was not statistically significant in either of the two ischemic cohorts.

An overall hypermethylation of *MMPs* and *TIMPs* genes was observed in plaque tissue samples, with a 65.7% of CpG up-methylated in the unstable portion of the plaque compared to the stable counterparts (Supplementary Figure 3A). The same behavior was detected when interrogating both blood-sample cohorts, with a 70.85% of CpGs up-methylated in case samples compared to atherothrombotic stroke patients in ISC-1 and 63.48% of the CpG up-methylated in ISC-2 (Supplementary Figure 3A). This trend was observed across all gene mapping compartments, mostly in the gene body and gene-TSS1500, and across all CpG islands regions, as well as in sites mapping into enhancer regions (Supplementary Figure 3B–3D).

## DISCUSSION

The role of MMPs in atherosclerosis has been extensively evaluated and their activity is essential for many processes involved in atherosclerotic plaque formation, such as infiltration of inflammatory cells, smooth muscle cell migration and proliferation and angiogenesis [11]. Furthermore, matrix degradation by MMPs causes plaque instability and rupture leading to unstable angina, myocardial infarction and stroke [12]. Müeller et al., identified increased expression levels of MMP1, MMP9, MMP12, and MMP14 in vulnerable plaques compared to stable ones, and lower expression levels in MMP2 and TIMP3 vulnerable plaques [5]. In contrast, Sapienza et al., observed an imbalance between MMPs and TIMPs plasma levels in unstable carotid plaques [6]. A recent multiancestry genome-wide-association meta-analysis [7] identified a locus in *MMP12* associated with atherothrombotic stroke, where the lead SNP was also associated with methylation and protein quantitative trait loci (meQTL and pQTL) [7].

In our study, unstable plaque samples had 65.7% of CpG up-methylated compared to their stable counterparts, indicating a trend to hypermethylation. These findings were most evident at the body and TSS1500 regions of the genes. Our results are in line with those of others, who observed a broad trend of DNA hypermethylation in plaque progression [8–10]. Zaina et al., observed pattern of genome-wide hypermethylation in atherosclerotic plaques, suggesting that an atherosclerosis-specific DNA methylation profile is established in the early stages of plaque evolution [9]. In a separate study, Zaina et al., identified small DNAm changes between symptomatic and asymptomatic plaques and an association with increasing post-cerebrovascular event time [8]. They observed a general hypermethylation in the plaque in early post-cerebrovascular event time compared to asymptomatic plaque. In another study that analyzed donor-matched atherosclerotic and normal aortic samples, the authors observed a correlation between histological grade and differential methylation between plaque pairs for 1,985 CpGs, most of which drifted toward hypermethylation with lesion progression [10].

MMP24 is a protease, member of the membrane-type MMP (MT-MMP), a subfamily in the matrix metalloproteinases-family, characterized by having a transmembrane domain and being expressed at the cell surface. Substrates of this protease include the proteins cadherin-2 and MMP2. Here, we present the first reported association between *MMP24* and atherosclerosis, although links with other MMPs in the MT-MMP subtype have been described before. For instance, MMP14, a member of the MT-MMP subfamily, has been implicated in acute myocardial infarction. Moreover, expression of *MMP14* can influence collagen content of mouse plaques, implying an important role for MMP14 in plaque stability [13]. While MMP24 was not assessed, a study reported, higher gene expression levels of *MMP14* in vulnerable plaques than stable plaques, (no other member of MT-MMP subfamily was interrogated) [5].

Our results indicate a distinct methylation state of the CpG site cg0431675 in *MMP24* between stable and unstable carotid plaque, which we have also detected associated with atherosclerotic stroke in two independent cohorts. A trend to hypermethylation is observed in unstable plaque-portion and non-stroke control samples, which falls into line with what has been postulated by others, i.e., that ruptured plaques tend to revert to a stable structure. Zaina et al., in their study, observed an initial hypermethylation in the transition from asymptomatic plaque to early post-cerebrovascular event time, followed by a reversion to initial methylation levels with increasing post-cerebrovascular event time, suggesting a process of plaque remodeling to a more stable phenotype [8]. Peeters W et al., proposed that ruptured plaques remodel to a relatively stable structure after stroke events [14]. They identified higher macrophage infiltration in carotid plaques, obtained from patients operated early after stroke, compared with asymptomatic patients, as well as significantly elevated levels of a set of proinflammatory cytokines and MMP8, MMP9, and MMP2 activity [14].

*TIMP2*, in addition to its role as metalloproteinase inhibitor, can directly suppress the proliferation of endothelial cells. *TIMP2* is a rather large gene, with 62 CpG sites of the 450k array mapping into the gene. Two CpG sites, in different regions of *TIMP2*, were statistically associated with plaque progression in our study, although this association was not observed between atherothrombotic stroke and control samples, either in statistical significance or directionally. Therefore, we cannot conclude much from those results, except that the most known activity of TIMP2 is MMP2 regulation [11]. It has been reported that human carotid plaque extracts promote platelet aggregation due to their MMP2 content, which can be inhibited by TIMP2, and that the ratio of MMP2/TIMP2 of plaques potentiating platelet aggregation is significantly higher than that of plaques not potentiating it. Moreover, an elevated MMP2 activity in plaques and a high aggregation-potentiating effect of plaques have been associated with a higher rate of subsequent ischemic cerebrovascular events [15].

Our results indicate that, there is a distinct methylation pattern of *MMPs* and *TIMPs* genes between stable and unstable carotid plaque, that might play a role in plaque instability. Three CpGs reached statistical significance, a bigger sample could have boosted the power of the study to identify other CpGs. While there is no previous literature linking our primary association, *MMP24*, with atherosclerosis, a vast catalog of studies on RNA expression, plasma levels studies, genetics and methylation [5–8, 13, 15] in the MMP gene family corroborate the biopathological plausibility of its role in plaque progression.

In summary, we characterized *MMPs* and *TIMPs* DNAm patterns in atheromatous plaque, which led us to observe significant differences between stable and ulcerated portions in plaque tissue for *MMP24* and *TIMP2*. Differences in *MMP24* were also observed in blood samples between atherothrombotic stroke patients and healthy controls. The generalized hypermethylation found in ulcerated portions and samples from healthy controls is in line with other methylation studies. Functional analysis of the implications of methylation levels changes in *MMP24* and *TIMP2* have in their expression levels are needed to link this finding with the biopathology of plaque destabilization. Studies with larger sample size, to confirm our results.

### Limitations

The study presents a small sample size, which might have limited the ability to find other potential biomarkers. Although other studies have also performed epigenetic experiments in a limited number of atherothrombotic plaques [10]. Using samples from the same patient decreases the variability, thus increasing the statistical power. Moreover, our blood results corroborate that *MMP24* may play a role in atherosclerosis due to epigenetic modifications. Additionally, having whole blood DNA samples derived from the same atheromatous plaque subjects would have given more insight into useful methylation biomarkers of plaque vulnerability.

## MATERIALS AND METHODS

### Human atherosclerotic plaque samples

Three atherosclerotic plaque samples were obtained at Hospital Universitari Mutua de Terrassa (Barcelona, Spain), according to a protocol approved by the local ethics committee. Patients with >70% stenosis underwent carotid endarterectomy surgery, following European Society for Vascular Surgery Guidelines [16]. Plaque sizes ranged from 1.5 to 3.5 cm and the samples were stored at –80°C until the analysis. Stable and ulcerated portions of plaque were macroscopically identified and sectioned prior to DNA extraction by a trained technician (Supplementary Figure 1). Sample selection was based on the size and definition of stable and ulcerated portions. Sample information for donor-matched plaque pairs is shown in Supplementary Table 1.

### Human whole blood samples

Methylation levels of MMPs and TIMPs in plaque tissue were crossed with methylation levels in blood tissue in two new independent cohorts. Ischemic stroke-cohort 1 (ISC-1): 37 atherothrombotic stroke patients and 6 healthy controls, from the GRECOS study [17]; Ischemic stroke-cohort 2 (ISC-2): 80 atherothrombotic patients from the BASICMAR prospective register [18] and 184 healthy controls from REGICOR population-based cohort (Supplementary Table 2). In both cases, only atherothrombotic stroke samples were selected as defined by the Trial of Org 10172 in Acute Stroke Treatment (TOAST) [19].

Local ethics committee approved the study (PR (AG) 03/2007). All patients signed a written informed consent.

### DNA preparation and bisulfite conversion

Genomic DNA was extracted from <10 mg of frozen plaque tissue using the QIAamp DNA Micro Kit (Qiagen, Hilden, Germany) tissue protocol. Genomic DNA, from fresh whole blood samples, was obtained using the Gentra Puregene Blood Kit (Qiagen, Hilden, Germany), following the manufacturer’s instructions, in the Plaque-tissue samples and the Blood-tissue ISC-1 samples. Blood-tissue ISC-2 samples were extracted using manual salt precipitation in Banco Nacional de ADN (Instituto de Salud Carlos III, Madrid, Spain).

### Methylation assays

Genome-wide DNAm was assessed using the Infinium HumanMethylation450 BeadChip (450K), in plaque and ISC-1 samples, and Infinium MethylationEPIC (EPIC) in ISC-2 samples, (Illumina Inc, San Diego, CA, USA).

### CpG site selection

Annotation information provided by Illumina’s 450K BeadChip manifest [20] was used to identify CpG sites (CpGs) mapped into the MMP and TIMP family of genes. A total of 27 genes (23 MMPs and 4 TIMPs) were identified, comprising a total of 464 CpGs, of which 415 passed quality controls (Supplementary Table 6).

### Data preprocessing, quality control (QC), and analysis

Functions from the ChAMP Bioconductor package v.2.9.19 [21] were used for data preprocessing, QC, normalization and univariate analysis. Following the ChAMP package pipeline recommendations, QC metrics were examined to determine the success of the bisulfite conversion and subsequent array hybridization. Fluorescence intensities were imported from GenomeStudio, then probe filtering was performed to remove probes that had failed to hybridize (detection *P* > 0.05) and were not represented by a minimum of 3 beads on the array. CpG sites containing documented single-nucleotide polymorphisms were also excluded. Multidimensional scaling plots were used to evaluate sex outliers based on chromosome X data. Multidimensional scaling and principal components were also used to check for unknown population structures. Probes mapping to X chromosome were not removed, so as not to rule out *TIMP1* CpG sites, mapped in the X chromosome. Recommended BMIQ method was used for data normalization. We also adjusted our data by cell proportion, with *champ. refbase* function.

Finally, Wilcoxon rank-sum test and the *Champ. DMP* function were used to identify differentially methylated positions among groups in the univariate model (Model_1), and *glm* for the multivariate models. Multivariate model, Model_2, included sex, age, smoking, variables, and Model_3 included sex, smoking, hypertension (HTA) and dyslipidemia (DL). This pipeline was used for the three cohorts, atherosclerotic plaque, ISC-1, and ISC-2, (except: ISC-2 cohort did remove the CpG sites mapped in the X chromosome).

### Statistical analysis and power calculation

The significance threshold, for all statistical tests, was set at False Discovery Rate (FDR) *p* < 0.05. Statistical analyses were performed using Bioconductor packages (http://www.bioconductor.org) and R software (http://www.cran.r-project.org).

As described by Tsai P et al., [22] we have an 80% power to detect *p* < 0.05, considering Cohen’s d effect size between 1.93–3.33 in the paired-plaque sample study and over 95% to detect *p* < 0.05, considering Cohen’s d effect size between 1.47–1.80.

## SUPPLEMENTARY MATERIALS





## Abbreviations

450K: Infinium HumanMethylation450 BeadChip
CpG: cytosine-phosphate-guanine
CpGs: CpG sites
CVD: Cardiovascular disease
DNAm: DNA methylation
DL: Dyslipidemia
EPIC: Infinium MethylationEPIC
FDR: False discovery rate
HTA: Hypertension
IS: Ischemic Stroke
ISC-1: Ischemic stroke cohort 1
ISC-2: Ischemic stroke cohort 2
meQTL: Methylation quantitative trait loci
MMP: Matrix Matalloproteinases
pQTL: Protein quantitative trait loci
QC: Quality control
TIMP: Tissue Inhibitor of Metalloproteinases
TOAST: Trial of Org 10172 in Acute Stroke Treatment
TSS1500: Transcription start site 1500
