# Supplementary material for: DNA methylation of MMPs and TIMPs in atherothrombosis process in carotid plaques and blood tissues
Source: Oncotarget. 2020 Mar 10;11(10):905–12. doi: 10.18632/oncotarget.27469 (PMC7075467; doi:10.18632/oncotarget.27469)
Supplement: Supplementary file 1 [file oncotarget-11-905-s001.pdf]

DNA methylation of MMPs and TIMPs in atherothrombosis process in carotid plaques and blood tissues

SUPPLEMENTARY MATERIALS

MATERIALS AND METHODS

Cohorts

Ischemic stroke-cohort 1 (ISC-1)

Thirty-seven (37) atherothrombotic ischemic stroke patients, as classified by the Trial of Org 10172 in Acute Stroke Treatment (TOAST), and six healthy controls, from the GRECOS study[1] (Supplementary Table 2) were selected.

The GRECOS cohort consisted of (n = 1494) consecutive Caucasian patients who presented with a first episode of persistent focal neurological deficit of less than 1 h (TIA) or more than 1 h with Neuroimaging (CT or MRI) confirming an ischemic stroke. Individuals who had previously been diagnosed with a stroke, those with mRS ≥ 4 at discharge and with a life expectancy of <1 year at the time of inclusion and patients participating in a clinical trial of secondary prevention of stroke were not included. Whole blood samples were obtained within the first 24 h after stroke onset, as described elsewhere[2]. Sample selection was based on the availability of DNA methylation data.

Ischemic stroke-cohort 2 (ISC-2)

80 atherothrombotic patients, and 362 healthy controls, from the BasicMar, were selected. The BasicMar Register prospectively recruited all consenting patients who were admitted to Hospital del Mar (Barcelona, Spain), from 2005 to 2012 (n = 4291; response rate, 80.8%) with a diagnosis of stroke fulfilling World Health Organization criteria. Inclusion criteria in BASICMAR cohorts were as follows: (1) first ischemic stroke; (2) brain imaging with CT or MRI; (3) availability of the clinical data supporting the assigned stroke subtype according to TOAST classification; (4) availability of the clinical data of HbA1c and BMI; and (5) absence of intracranial hemorrhage, neoplasms, demyelinating and autoimmune diseases and vasculitides. All patients were assessed and classified by a neurologist and were included in the study by consecutive order of recruitment.

All patients or their relatives were provided with oral and written information and signed the informed consent.

Supplementary Table 1: Descriptive characteristics of atherosclerotic plaque samples from donor patients at Hospital Universitari Mutua de Terrassa

Sample	Age	Gender	Current Smoker	Hypertension	Diabetes Mellitus	Dyslipidemia	Atrial Fibrillation	Ischemic Stroke
Plaque_1	77	M	No	Yes	No	Yes	Yes	Yes
Plaque_2	65	M	No	Yes	No	Yes	NA	No
Plaque_3	69	M	NA	NA	NA	NA	NA	No

Supplementary Table 2: Descriptive characteristics and bivariate analysis of atherothrombotic ischemic stroke patients and controls from the GRECOS study

	Ischemic Stroke Cohort 1			Ischemic Stroke Cohort 2		
	**Atherothrombotic stroke**	**Control**	***p*-Value**	**Atherothrombotic stroke**	**Control**	***p*-Value**
**N**	37	6	-	80	184	-
**Age, years**[¥]	68.2 (9.6)	71.5 (9.3)	0.3	70 (12.1)	63.4 (6.9)	<0.001
**Sex, male, *n* (%)**	32 (91.4)	5 (83.3)	0.89	64 (79)	88 (47.8)	<0.001
**Presence of Dyslipidemia**	18 (19.4)	2 (33.3)	0.71	43 (53.8)	124 (67.8)	0.039
**Presence of Diabetes Mellitus**	11 (31.8)	1 (16.7)	0.80	29 (35.8)	31 (16.8)	0.001
**Presence of Hypertension, *n* (%)**	26 (13.5)	6 (100)	0.38	65 (80.2)	208(58.7)	0.001
**Current Smoker, *n* (%)**	9 (38.9)	1 (16.7)	0.79	37 (46.3)	16 (8.7)	<0.001
**Statins, yes, *n* (%)**	24 (14.6)	0 (0)	4.83E-04	-	-	-

[¥]*Mean (standard deviation).*

Supplementary Table 3: Results from the association study between atherothrombotic ischemic stroke patients and controls, using whole blood tissue. ISC-1 represents the ischemic cohort one, and ISC-2 the ischemic cohort two. Data is sorted in ascending order, based on ISC-2 *p*-values, *NA* values in ISC-2 are from CpGs that were not in ISC-2. See Supplementary Table 3

Supplementary Table 4: Differentially methylated CpG sites between normal and ulcerated plaque-portions

CpG	Gene	Chr	Position	Mapping to genes	Mapping to cgi	Enhancer	Average methylation Δβ-value	*p*-Value	*q*-Value
cg24211657	*TIMP2*	17	76886742	Body	opensea	TRUE	-0.159	9.48E-05	0.036
cg02969624	*TIMP2*	17	76849181	3’UTR	shelf	TRUE	0.157	1.71E-04	0.036
cg04316754	*MMP24*	20	33815109	Body	island	TRUE	0.079	2.70E-04	0.037

Positive average delta beta-values (*Δβ-values*) values represent a shift towards up-methylation in ulcerated plaque portions, whereas negative values represent a shift towards down-methylation. FDR corrected *p*-values (*q*-values < 0.05), regular letters represent paired *t*-test *p*-value < 0.05).

Supplementary Table 5: Results from our association study in Ischemic Stroke Cohort 2 (ISC-2) blood samples, using Model_1 (univariate analysis) and Model_3 (glm- adjusted by gender, smoking, HTA and DL)

		Model_1	Model_3
**cg02969624**	*TIMP2*	9.57E-01	3.52E-07
**cg04316754**	*MMP24*	1.90E-04	3.1E-07

Model_3 represents the fittest model, for our CpGs of interest.

Supplementary Table 6: Number of CpG sites annotated in each *MMP* and *TIMP* genes, before and after quality controls (QC), for each cohort

	N CpGs			
**Gene**	**pre-QC**	**Plaque post-QC**	**ISC-2 post-QC**	**ISC-1 post-QC**
MMP1	7	7	7	7
MMP2	22	16	15	16
MMP3	7	7	5	7
MMP7	7	7	7	7
MMP8	2	1	1	1
MMP9	20	20	14	20
MMP10	2	2	2	2
MMP11	13	13	10	13
MMP12	6	5	4	5
MMP13	10	9	9	9
MMP14	22	21	20	21
MMP15	38	36	32	36
MMP16	29	27	23	27
MMP17	58	50	43	50
MMP19	7	6	6	6
MMP20	7	6	2	6
MMP21	21	14	13	14
MMP23	12	3	3	3
MMP24	8	6	6	6
MMP25	22	22	19	22
MMP26	7	5	5	5
MMP27	5	4	3	4
MMP28	28	25	20	25
TIMP1	10	9	0	9
TIMP2	65	64	55	64
TIMP3	22	20	0	20
TIMP4	10	10	3	10
**TOTAL N.**	467	**415**	**355**	**415**

Annotation information provided by Illumina's 450K BeadChip manifest[3].

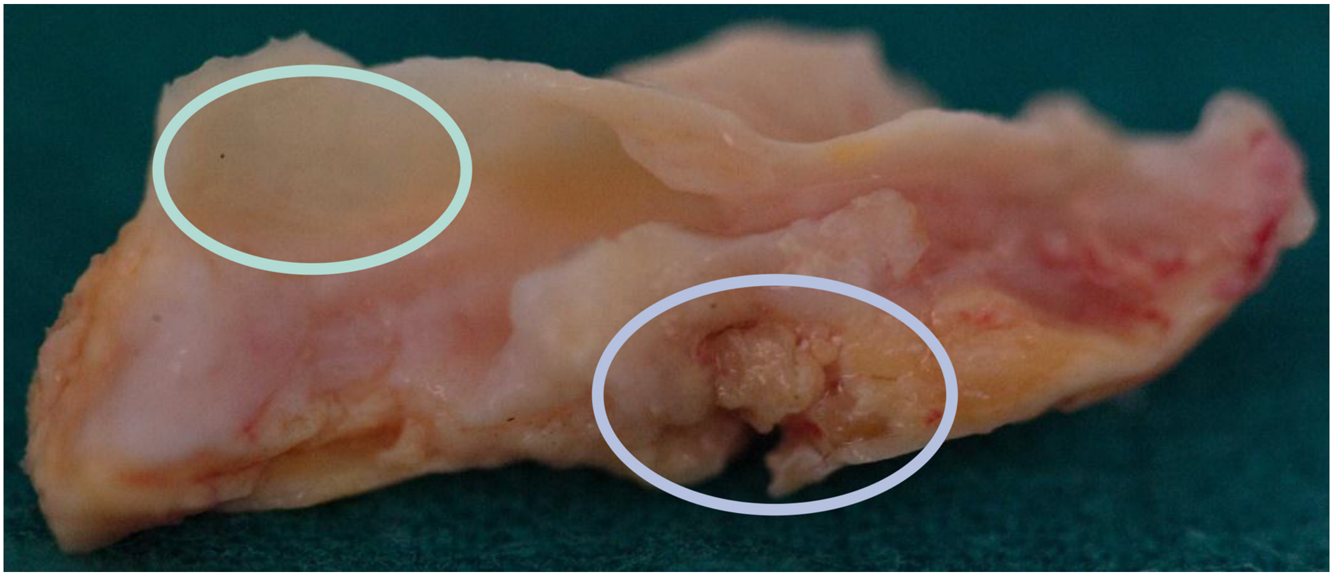

Supplementary Figure 1: Macroscopic image of sample *Plaque_1*. Green and Blue sections were macroscopically identified as stable (Stable_Plaque_1) and ulcerated (Unstable_Plaue_1) plaque portions, respectively.

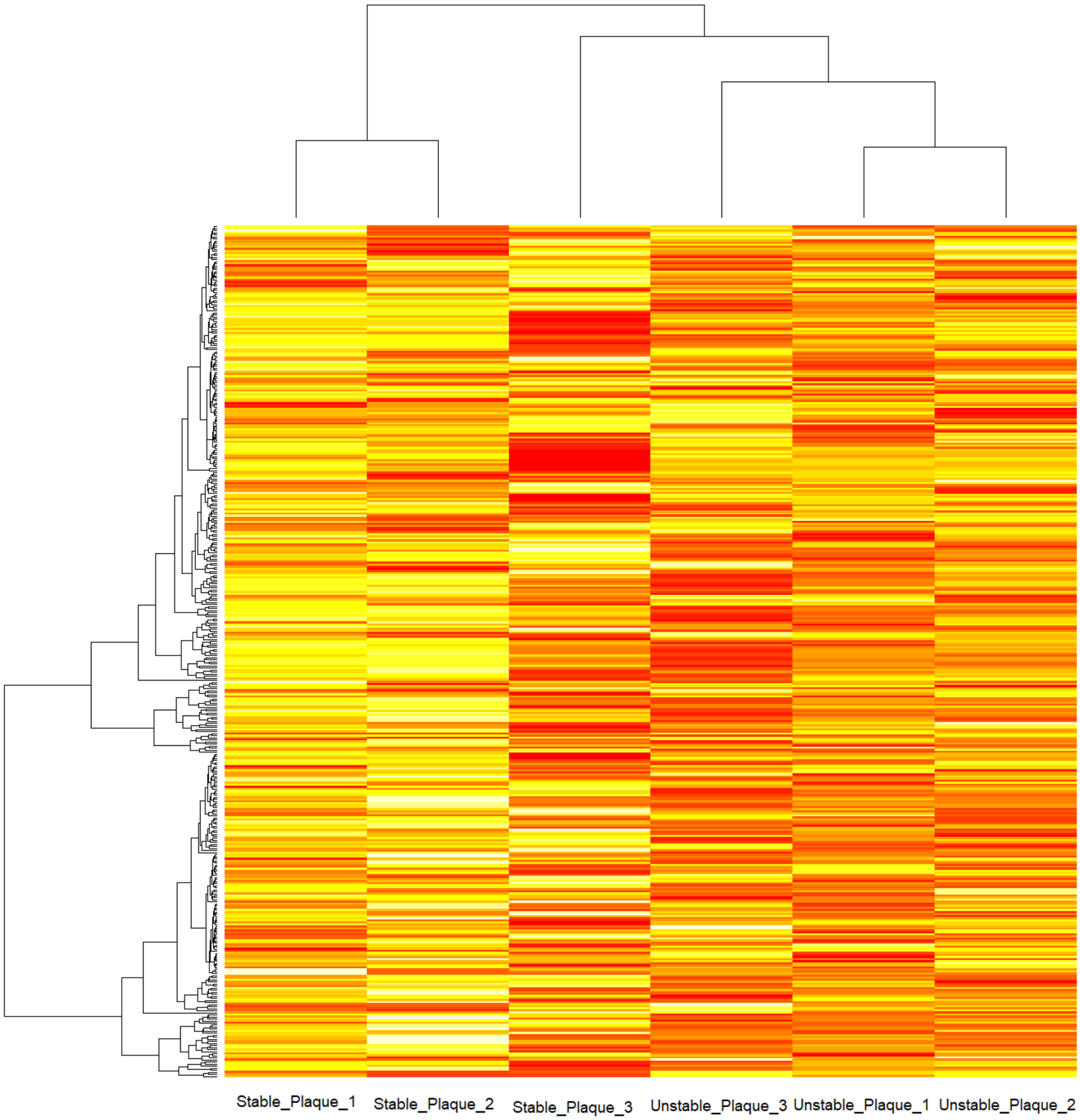

Supplementary Figure 2: Cluster analysis of donor-matched aortic plaque samples. Supervised clustering of all 415 CpGs. Notice almost perfect segregation between stable and unstable plaque counterpart, only one sample is misplaced. Columns represent samples, horizontal lines represent CpGs.

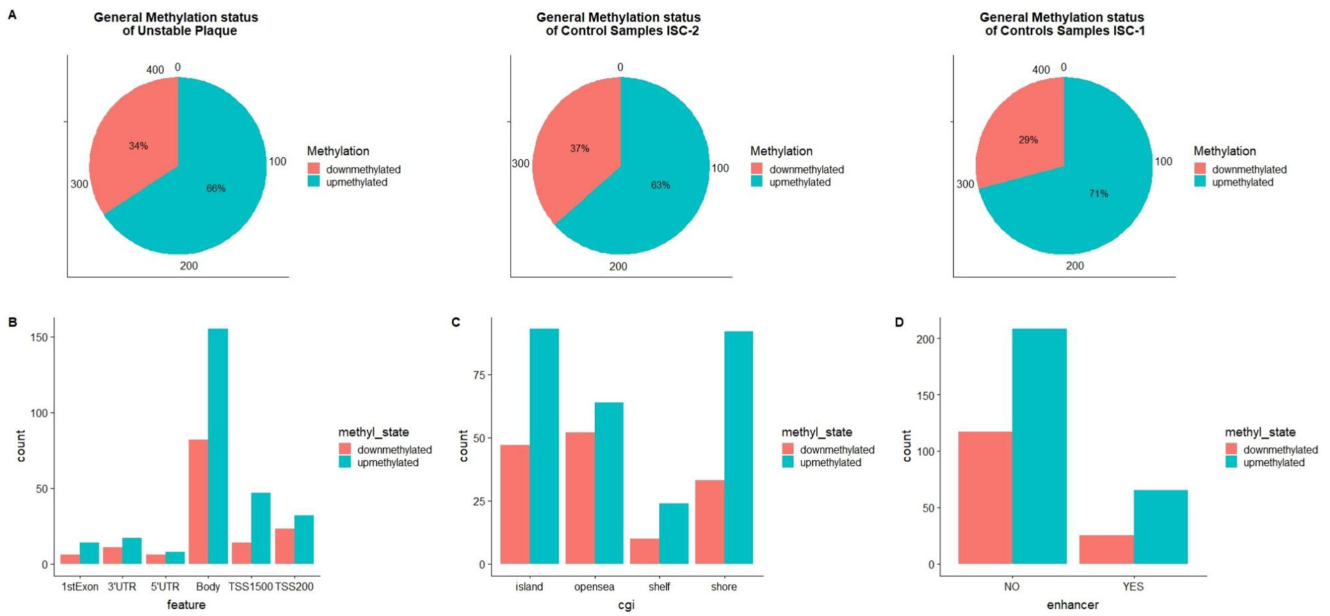

Supplementary Figure 3: General methylation status of MMPs and TIMPs, and it's genomic mapping. (**A**) Percentage of up- and down-methylated enhancers; (**B**) Mapping relative to gene compartments, (**C**) and CpG islands (CGI), (**D**) proportion of up- and down-methylated enhancers.
